# Unraveling molecular networks in thymic epithelial tumors: deciphering the unique signatures

**DOI:** 10.3389/fimmu.2023.1264325

**Published:** 2023-10-02

**Authors:** Xiao Zhang, Pengpeng Zhang, Ansheng Cong, Yanlong Feng, Hao Chi, Zhijia Xia, Hailin Tang

**Affiliations:** ^1^ State Key Laboratory of Oncology in South China, Sun Yat-Sen University Cancer Center, Guangzhou, China; ^2^ Department of Thoracic Surgery, The First Affiliated Hospital of Nanjing Medical University, Nanjing, China; ^3^ Department of Lung Cancer Surgery, Tianjin Medical University Cancer Institute and Hospital, Tianjin, China; ^4^ Division of Nephrology, Nanfang Hospital, Southern Medical University, State Key Laboratory of Organ Failure Research, Guangdong Provincial Key Laboratory of Nephrology, Guangzhou, China; ^5^ School of Clinical Medical Sciences, Southwest Medical University, Luzhou, China; ^6^ Department of General, Visceral, and Transplant Surgery, Ludwig-Maximilians University Munich, Munich, Germany

**Keywords:** thymic epithelial tumors, thymoma, thymic carcinoma, molecular, genomic, cellular signaling network

## Abstract

Thymic epithelial tumors (TETs) are a rare and diverse group of neoplasms characterized by distinct molecular signatures. This review delves into the complex molecular networks of TETs, highlighting key aspects such as chromosomal abnormalities, molecular subtypes, aberrant gene mutations and expressions, structural gene rearrangements, and epigenetic changes. Additionally, the influence of the dynamic tumor microenvironment on TET behavior and therapeutic responses is examined. A thorough understanding of these facets elucidates TET pathogenesis, offering avenues for enhancing diagnostic accuracy, refining prognostic assessments, and tailoring targeted therapeutic strategies. Our review underscores the importance of deciphering TETs’ unique molecular signatures to advance personalized treatment paradigms and improve patient outcomes. We also discuss future research directions and anticipated challenges in this intriguing field.

## Introduction

Thymic epithelial tumors (TETs) are an uncommon group of neoplasms located primarily in the anterior mediastinum, representing only a fraction of approximately 0.2-1.5% of all malignant tumors, with an annual incidence ranging from 1.3 to 3.2 cases per million (1, 2). TETs exhibit a remarkable histological heterogeneity, as classified by the World Health Organization in 2021, including thymomas (types A, AB, B1, B2, B3) and thymic carcinomas (type C) (3). Thymomas are generally characterized as low or intermediate-grade malignancies, boasting a favorable 5-year overall survival (OS) rate exceeding 80% (4). In contrast, thymic carcinomas exhibit increased invasiveness, metastatic potential and a higher propensity for recurrence, resulting in a worse 5-year overall survival rate of approximately 36% (5). Radical surgical resection remains the gold standard treatment strategy for early-stage TETs, while advanced or metastatic cases necessitate a multimodal approach integrating surgery with radiochemotherapy (6). Nevertheless, it is worth noting that only a small minority of patients with unresectable thymic epithelial tumors demonstrate any response to radiochemotherapy (7), and the implementation of targeted therapies and immunotherapies remains a major challenge in the clinical management of these malignancies. Due to the rarity of TETs, their clinical and biological heterogeneity, alterations in histopathological classification, and the dearth of well-established cell lines and animal models, the molecular investigation of TETs is fraught with significant challenges.

Nevertheless, recent advancements in molecular biology techniques have propelled the discovery of extensive molecular alterations and perturbations in signal transduction pathways within TETs. These insights have significantly enriched our comprehension of the molecular mechanisms driving tumorigenesis and accelerated progress in the development of systemic treatments for TETs. The exploration of the molecular landscape of TETs is of paramount importance for various reasons. Primarily, it offers a conduit to uncover the intricate mechanisms of tumorigenesis, thereby refining our understanding of the pathophysiology of these uncommon tumors. Further, it holds promise in enhancing diagnostic and prognostic evaluations, thereby serving as a foundation for personalized patient care. Importantly, deciphering the complex molecular architecture of TETs could steer the development of precision therapeutic strategies, possessing the potential to improve treatment outcomes and patient prognosis.

**Figure 1:**
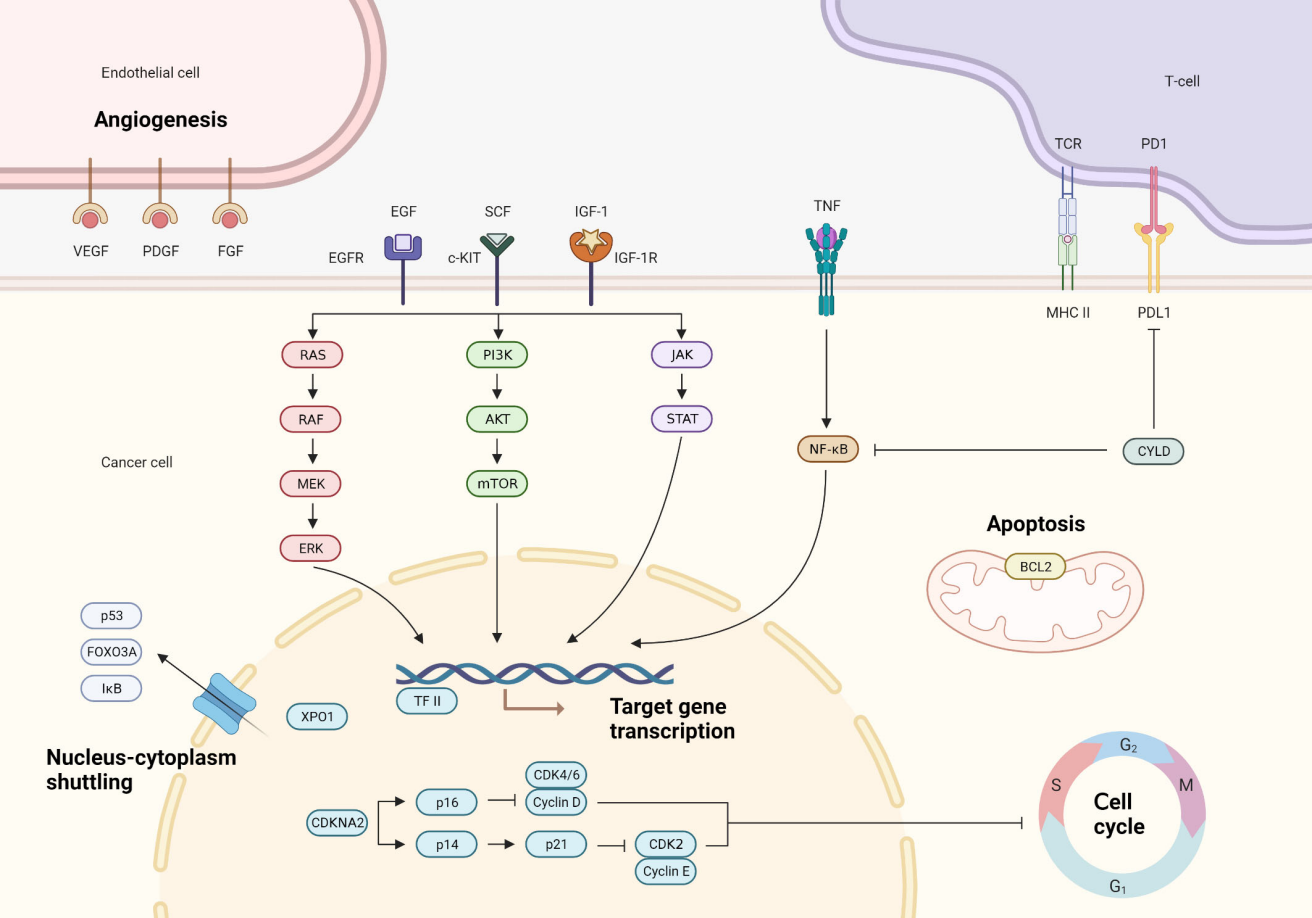
Main molecular pathways involved in the pathogenesis of thymic epithelial tumors.

In this comprehensive review, we navigate through the complex molecular terrain of TETs (**Figure 1**). We begin by analyzing chromosomal abnormalities and their role in disrupting key genes and signaling pathways integral to tumorigenesis. Subsequently, we explore the molecular subtypes of TETs, discussing their implications for tailoring therapeutic approaches. The focus then shifts to the implications of gene mutations and aberrant gene expressions on disease progression. We also examine the influence of structural gene rearrangements and epigenetic deviations in shaping TET pathogenesis. Finally, we underscore the significant impact of the tumor microenvironment on tumor behavior and response to therapy. Our goal is to provide an in-depth understanding of the molecular networks within TETs, illuminating potential pathways for enhancing diagnostic precision, improving prognostic accuracy, and advancing therapeutic strategies.

## Chromosome abnormalities

In the landscape of TETs, a vast majority of chromosomal alterations continue to hold uncertain biological implications. Observations indicate that arm-level copy number losses are most frequently identified on chromosome 6, with a prevalence of 26% at 6p and 29% at 6q. These chromosomal losses are followed in frequency by those on 3p (22%) and 13q (18%). Simultaneously, the most recurrent arm-level copy number gains are found to affect chromosomes 1q (55%), 7p (20%), 7q (15%), and 20p (17%) (8). The biological consequences of these alterations, however, require further exploration. It’s noteworthy that chromosomal copy number aberrations appear with increased frequency in histological subtypes typified by heightened invasiveness. Reports suggest that type A thymomas exhibit a comparatively lower spectrum of copy number aberrations. This stands in stark contrast to the recurrent observation of arm-level copy number increase on 1q and decrease on chromosome 6 in type B2/B3 thymomas. Type B3 thymomas and thymic carcinomas display a copy number aberration profile that echoes that of type B2/B3 thymomas. However, they deviate in terms of the copy number losses on chromosomes 13q, 16q, and 17p, which are conspicuously absent in type B2/B3 thymomas (9). The precise biological significance of these alterations in TETs remains largely elusive, with much yet to be determined in the vast majority of cases.

However, it is noteworthy that TETs exhibiting copy number gains in B-cell lymphoma-2 (BCL2) and loss of cyclin-dependent kinase inhibitor 2A/B (CDKN2A/B) are consistently associated with an unfavorable prognosis. BCL2, known for its involvement in apoptosis regulation, has been extensively demonstrated to contribute to tumorigenesis, as gene amplification disrupts apoptosis and promotes tumor cell accumulation. Notably, the oncogenic role of BCL2 is well-established in follicular lymphomas, where the juxtaposition of IgH-BCL2 is implicated in lymphomagenesis, perturbing normal B lymphocyte apoptosis (10). Beyond their established role in apoptosis regulation, members of the BCL2 family are also implicated in the control of autophagy and necroptosis pathways. Amplification of the BCL2 gene locus has been identified in around 10% of TET cases. Furthermore, copy number gains of MCL1, another member of the BCL2 family gene, have been observed in 51% of TETs, predominantly in aggressive histological subtypes (83% in type B2/B3 thymomas, 70% in type B3 thymomas and 57% in thymic carcinomas). It is worth noting that these MCL1 copy number gains are mostly attributed to the broad gain of 1q, rather than focal MCL1 amplification (9). The concurrent expression of MCL1 and BCL2 is frequently observed in aggressive TETs, thereby suggesting their potential role as prognostic indicators (11). *In vitro* studies provide compelling evidence that the suppression of BCL2 family proteins, including MCL1 and BCL-XL, precipitates apoptosis in TET cell lines. These findings hint at the possibility that therapeutic strategies aimed at the dysregulated anti-apoptotic BCL2 family proteins could provide a viable avenue for TET treatment (12). Loss of chromosome 6q25.2–25.3 is observed across all subtypes of thymomas, with the exception of type B1 thymomas. This genetic alteration has been associated with a decrease in the expression of the FOXC1 protein, which is encoded by the tumor suppressor gene FOXC1 situated within the 6p23 locus. Notably, individuals presenting with a FOXC1 deficiency display an expedited rate of tumor progression, as well as a markedly abbreviated disease-related survival span (13). *In vitro* findings lend credence to the potential tumor suppressor activity of FOXC1, particularly in its impact on cell proliferation (14).

## Molecular subtypes

In recent years, the Cancer Genome Atlas (TGCA) has conducted extensive research on TET using multi-platform next-generation sequencing (NGS). This research has yielded a more comprehensive understanding of the molecular landscape of TET and has identified four distinct molecular subtypes. Among these, Subtype 1 is predominantly associated with type B thymoma and has a notable prevalence of tumors associated with myasthenia gravis. Subtype 2, on the other hand, is primarily associated with thymic carcinoma and exhibits a higher tumor mutation burden (TMB), upregulation of oncogenes, downregulation of tumor suppressor genes, and deletion of chromosome 16q. Subtype 3 is characterized by type AB thymoma and is distinguished by a high prevalence of GTF2I mutations and abundant lymphocytic infiltration. Finally, Subtype 4 includes both type A and type AB thymomas and displays a relatively higher incidence of somatic mutations in GTF2I and HRAS. These subtypes exhibit distinct molecular features and are highly consistent with the histological classification by the World Health Organization (WHO), thereby confirming the non-continuous nature of TET in terms of its biological entities (15). Another investigation, leveraging the rich dataset of TCGA, has compellingly demonstrated the feasibility of classifying TET into four distinct groups: the GTF2I mutation group, the T-cell signaling (TS) group, the chromosomally stable (CS) group, and the chromosomally instability (CIN) group. The molecular taxonomy correlates with the clinical and histological characteristics. For instance, The GTF2I subtype aligns with a more favorable WHO histology, earlier Masaoka-Koga staging, and absence of myasthenia gravis (MG), while the CIN subtype exhibits less advantageous WHO histology, advanced Masaoka-Koga staging, and the presence of MG. The prognostic potential of this molecular classification system is elucidated by its capacity to reconcile the heterogeneity in clinical outcomes within identically classified patient cohorts. Moreover, this molecular classification system serves as a robust tool for discerning therapeutic responsiveness. Tumors within the TS group, enriched with genes implicated in co-stimulatory and co-inhibitory T cell signaling pathways, such as PD1, may exhibit heightened responsiveness to immune checkpoint inhibitor (ICI) treatments. The CIN molecular subtype is characterized by the activation of EGF or SRC-FAK signaling pathways, while the CS group tumors exhibit an abundance of genes associated with epithelial-mesenchymal transition (EMT) pathways and Toll-like receptor signaling (16). Current investigations have corroborated the therapeutic potential of drugs targeting these specific molecular points within TETs (17–20). Indeed, the management of highly heterogeneous tumors, such as advanced hepatocellular carcinoma and TETs, poses a formidable challenge in the quest for universally effective therapies. However, the establishment of molecular classifications has significantly enhanced our understanding of these malignancies, paving the way for prospective clinical investigations centered around these distinct subtypes. Such pursuits hold paramount importance as they hold the potential to unlock personalized treatment strategies, tailored to the unique characteristics of individual patients, thus optimizing the efficacy of targeted therapeutic interventions (15, 16, 21, 22).

## Gene mutation abnormalities

The scientific community has recently gained advanced insights into the dynamics of gene alterations within thymic epithelial tumors (TETs). This progress parallels advancements in other tumor entities and is largely attributable to the innovative strides made in next-generation sequencing technologies. A defining characteristic of TET genomes is the concentration of C > T mutations in CpG islands, a mutation signature intrinsically linked with aging and congruent with the median age of disease onset. Insights gleaned from extensive genomic analyses within TCGA initiative indicate that distinct molecular modifications are a hallmark of various histological TET subtypes (15). Generally, the molecular aberration patterns of thymomas and thymic carcinomas are markedly different, with only a select few genes demonstrating significant and periodic mutations. A point mutation p. (Leu404His) in the general transcription factor II-i (GTF2I) gene, which has thus far remained elusive in other tumor types, has been identified with notable frequency within thymomas. On the other hand, thymic carcinomas bear a higher load of recurrent mutations within recognized cancer-associated genes, including but not limited to TP53, Cylindromatosis (CYLD), CDKN2A, BRCA1 associated protein 1 (BAP1), and polybromide 1 (PBRM1) (8).

TMB serves as a quantitative measure of the genomic alterations occurring within tumor cells, representing a biological marker for the emergence of novel antigens. Elevated TMB amplifies the capacity of the tumor to provoke immune responses, thereby enhancing the likelihood of benefiting from immunotherapeutic interventions (23). Among human malignancies, TETs stand out as one of the neoplastic entities with the lowest TMB. Notably, TMB exhibits a substantial increase in thymic carcinoma samples (3.84 mutations/Mb) when compared to thymomas (1.92 mutations/Mb) (24). Significantly, TMB exhibits a robust correlation with advanced clinical staging, more advanced pathological types, and patient age progression (8).

GTF2I mutations emerge as dominant genetic abnormalities in the context of TETs, highlighting their importance within the complex landscape of tumorigenesis. Of note, these mutations exhibit a discernible predilection for type A (100%) and type AB (70%) thymomas, with a modest but observable incidence detected in thymic carcinomas (8%). Interestingly, the mutational events are primarily localized to the codon L424H, reflecting the characteristic signatures of oncogenic mutation. Remarkably, the specificity of GTF2I mutations becomes apparent when scrutinizing other malignancies, where their occurrence remains a rarity (<1%) and consistently arises at distinct genomic sites apart from L424H. Patients with tumors characterized by GTF2I mutations exhibited a significantly more favorable prognosis when compared to those harboring wild-type GTF2I, as evidenced by a remarkable disparity in 10-year overall survival (96% versus 70%). Mechanistically, GTF2I encodes the pivotal TF2I protein, whose orchestrated activation in the cellular nucleus imparts profound influence on the transcriptional machinery that controls key biological processes encompassing cell cycle progression, DNA repair, cell proliferation, and the intricate modulation of TSC/mTOR signaling cascades. Pertinently, the disruptive L424H missense mutation impairs the normal degradation kinetics of TF2I protein, culminating in its intracellular accumulation. Subsequently, this dysregulation sets in motion a cascade of molecular events leading to discernible alterations in the gene expression landscape. Notably, genes associated with cell morphogenesis, receptor tyrosine kinases, retinoic acid receptors, and WNT/SHH signaling pathways exhibit augmented expression levels, while those involved in cell cycle regulation, apoptosis, DNA damage response, hormone receptor signaling, RAS/MAPK, and mTOR pathways manifest dampened expression levels (8, 15).

To gain deeper insights into the realm of GTF2I-mutant thymomas, Y. He et al. successfully engineered a murine model recapitulating the intricacies of GTF2I mutations, thereby successfully instigating thymoma formation in aged mice. Impressively, the resultant mouse thymomas mirrored key cellular features reminiscent of human type B1 and B2 thymomas at the transcriptional level. Remarkably, a substantial fraction of thymoma lesions exhibited a conspicuous enrichment in gene signatures associated with cortical thymic epithelial cells (cTECs) and thymic epithelial progenitor cells (TEPCs), closely resembling the enrichment patterns observed in GTF2I-mutant human TETs. Furthermore, our investigation unveiled an elevated abundance of cTECs and TEPCs within the thymic tissues isolated from mouse thymic tissue, thereby suggesting a potential cellular origin of GTF2I-mutant thymomas from these distinct cell populations. Mechanistically, aberrant activation of cell cycle-associated pathways mediated by MYC and E2F signaling cascades likely serves as a crucial driving force behind the initiation and development of GTF2I-mutant thymomas (25). Impressively, an investigation into micronodular thymoma with lymphoid stroma revealed a consistent occurrence of the GTF2I p.L424H mutation across all twelve sampled thymoma specimens, establishing the GTF2I p.L424H mutation as an invariable genetic hallmark of micronodular thymomas with lymphoid stroma. These findings compellingly infer a profound correlation between micronodular thymomas with lymphoid stroma and their type A and AB counterparts (26).

Activation of the PI3K/AKT/mTOR signaling pathway plays a critical role in driving the proliferation of TETs. The PI3K/AKT/mTOR signaling pathway is a critical orchestrator of numerous cellular processes, encompassing proliferation, survival, metabolism, and angiogenesis. Its dysregulation is frequently observed in a myriad of cancer types (27). Genetic aberrations at various levels of this pathway, including PI3K, AKT, TSC, and mTOR, have been identified within the context of TET.A gene mutation affecting the regulatory subunit of PI3K has been identified in a thymic carcinoma cell line. Employing NGS analysis on a cohort comprising 54 TET samples, three distinct mutations have been elucidated across three samples, impacting either the catalytic or regulatory subunits of the PI3K gene. Notably, compelling evidence from *in vitro* investigations demonstrates the potent anti-tumor efficacy of PI3K inhibitors, particularly within cellular populations harboring mutations in the PI3K gene (28). An additional study delved into the expression patterns of Akt, mTOR, and P70S6K in type A, type B, and type AB thymomas, substantiating the perturbed Akt/mTOR pathway in these thymomas. Moreover, the anti-proliferative impact of the mTOR inhibitor rapamycin on thymic epithelial cells underscored the significance of the Akt/mTOR pathway as a pivotal mechanism in tumorigenesis, thereby presenting a promising target for pharmacological intervention (29). *In vitro* studies unveiled that the silencing of the anti-apoptotic molecule BCL2 via siRNA knockdowns led to diminished cellular proliferation, while *in vivo* administration of pan-BCL2 inhibitors elicited notable suppression of xenograft growth, with mechanistic implications involving the PI3K/AKT/mTOR pathway (9). In addition, the activation of PIK3 has been validated to be linked with the overexpression of microRNA clusters located on chr19q13.42 in type A and type AB thymomas (30). In a single-arm phase II trial, researchers evaluated the efficacy of Everolimus in 50 patients with TETs, previously treated with cisplatin-based regimens. A disease control rate (DCR) of 88% was recorded, with a median progression-free survival of 10 months (16.6 months for thymomas and 5.6 months for thymic carcinomas). Indeed, mirroring observations in other solid tumors, the benefits of Everolimus appeared predominantly tied to disease stabilization or minor tumor shrinkage. Only 10% of patients experienced partial remission, and a mere 2% reported complete remission. Safety remained a concern throughout the trial, as 14 patients experienced severe drug-related adverse events (AEs), with 3 thymoma patients succumbing to drug-related pneumonia (31). Likewise, a clinical trial using the pan-PI3K inhibitor buparlisib demonstrated modest activity in B2 and B3 thymomas but was marred by the necessity for early drug cessation in over half the patients due to skin and lung toxicity (32). This underscores the need for extreme caution when considering PI3K inhibitors for use in TETs.

TETs frequently harbor mutations within the RAS family, with thymic carcinoma exhibiting a markedly higher incidence (24.1%) than thymoma (12.1%) (33, 34). Notably, NRAS mutations predominantly manifest in thymic carcinoma, whereas HRAS mutations are more prevalent in thymoma cases. The vast majority of mutations in HRAS and NRAS were found to be localized at recognized gain-of-function codons. Notably, HRAS mutations were predominantly detected at codons 12, 13, and 117, while NRAS mutations were primarily observed at codon 61. GTF2I, HRAS, NRAS, and TP53 have emerged as plausible candidates for initiating mutations in tumorigenesis or early stages of tumor development (15).

TP53 stands as one of the most prevalent genes with mutations, found in approximately 25% of thymic carcinoma cases and 5% of thymoma cases (8). Patients harboring TP53 mutations experience worse prognoses and demonstrate lower overall survival rates (35, 36). The TP53 gene encodes the crucial tumor suppressor protein p53, which assumes a significant role in thymic physiology. Within normal thymic cells, p53 acts as a regulator, facilitating the expression of RANK (receptor activator of nuclear factor kappa-B) and orchestrating the differentiation of medullary epithelial cells. Notably, p53 exhibits remarkable specificity in transcriptional control, specifically targeting genes associated with the function of medullary epithelial cells. Disruption or loss of p53 function yields profound consequences for thymic ontogeny, perturbing the delicate equilibrium of T cell homeostasis and compromising immune tolerance (37).

The tumor suppressor gene CDKN2A undergoes a process of selective splicing, resulting in the production of two distinct protein isoforms, namely p16INK4A and p14ARF. The functional role of p16INK4A lies in its ability to impede the progression of the cell cycle through targeted inhibition of cyclin-dependent kinases CDK4 and CDK6. On the other hand, p14ARF functions as an activator of the tumor suppressor TP53. Perturbations in CDKN2A can potentially instigate the activation of cyclin-dependent kinases, thereby disrupting the finely tuned regulation of the cell cycle (38). The occurrence of CDKN2A mutations has been reported in approximately 11% of thymic carcinomas (33), demonstrating an association with diminished overall survival rates compared to wild-type counterparts. Notably, a strong correlation has been observed between homozygous deletion of CDKN2A and loss of p16 expression. This correlation is particularly pronounced among younger patients with the squamous cell carcinoma subtype and is linked to unfavorable prognostic outcomes. Encouragingly, CDK4/6 inhibitors represent a promising therapeutic approach for CDKN2A-mutated cancers, including thymic carcinoma (39). Within the context of a multicenter, single-arm phase II trial, an ensemble of 48 patients, all grappling with recurrent or metastatic advanced TETs unresponsive to platinum-based chemotherapy, were administered monotherapy with palbociclib, yielding results that were decidedly heartening. The median duration of the follow-up was 14.5 months, and the objective response rate (ORR) stood at 12.5% (four instances of partial response in thymomas and two in thymic carcinomas). The progression-free survival (PFS) at six months was noted at 60.2%, with a median PFS of 11.0 months. Furthermore, the median overall survival span was registered at 26.4 months (40).

## Gene expression abnormalities

The expression of insulin-like growth factor receptor 1 (IGF-1R) is more frequently observed in histological subtypes characterized by a higher degree of invasiveness. Notably, a significant proportion (86%) of thymic carcinomas display moderate to high levels of IGF-1R expression, which is closely associated with epidermal growth factor receptor (EGFR) overexpression. Functionally, IGF-1R plays a pivotal role in processes such as tumorigenesis and resistance to EGFR inhibitors through the formation of heterodimers with EGFR, known as EGFR/IGF-1R heterodimers (41, 42). In a phase 2 clinical trial conducted to assess the therapeutic efficacy of cixutumumab, a monoclonal antibody targeting the IGF-1R, notable findings were obtained. Among the cohort of 37 patients diagnosed with thymomas, a partial response (PR) was observed in 14% of cases, while stable disease (SD) was achieved in 76% of cases. In contrast, within the subset of 12 patients with thymic carcinomas, no cases of partial response were observed, with only 5 cases demonstrating stable disease. Importantly, it is worth noting that approximately 24% of thymoma patients experienced the onset of autoimmune disorders during the treatment period. The therapeutic application of IGFR inhibitors is hindered by their pronounced toxicity profile, thus posing challenges in the management of diverse malignancies (43).

EGFR pathway mutations have emerged as independent prognostic factors significantly associated with reduced OS (34). Immunohistochemical analysis has revealed EGFR overexpression in 70% of thymomas and 53% of thymic carcinomas, with higher EGFR staining levels demonstrating a notable correlation with advanced stage III to IV tumors (44). Notably, EGFR mutations are exceedingly rare occurrences, and although EGFR amplification has been observed in B3-type thymomas, its association with EGFR expression remains elusive (45). The utilization of anti-EGFR agents, including cetuximab and erlotinib, has been documented in isolated cases (46, 47). However, the efficacy of combined treatment with erlotinib and bevacizumab in thymic carcinoma exhibits limited activity (48). The overexpression of the HER2 protein is frequently observed in thymic carcinomas, with HER2 expression detected in 58% of squamous cell thymic carcinomas. However, HER2 gene amplification is a rare occurrence, thus limiting the utility of HER2 as a viable therapeutic target in the context of thymic carcinomas (49, 50).

Aberrant neovascularization plays a pivotal role in the pathogenesis of TETs, exhibiting a compelling correlation with their invasive nature. Notably, thymic carcinoma outperforms thymoma in terms of promoting aggressive angiogenesis (51). A wealth of empirical evidence substantiates the conspicuous upregulation of vascular endothelial growth factor (VEGF), platelet-derived growth factor (PDGF), and other key angiogenic factors and receptors in TETs, showcasing notable associations with histologically distinct invasive phenotypes (52–54). Activin A, a valued member of the transforming growth factor Β (TGF-Β) superfamily, orchestrates cellular responses by downregulating the expression of p21 and VEGF, thereby eliciting a notable suppressive impact on endothelial cell proliferation. Conversely, Follistatin, acting as an antagonistic counterpart to activin A, effectively counteracts its growth-inhibitory and pro-apoptotic consequences. Moreover, Follistatin demonstrates a remarkable capacity to bind and activate angiogenic factors, thereby stimulating robust angiogenesis. Clinical studies have unveiled a conspicuous elevation in serum concentrations of activin A and Follistatin among TET patients, surpassing those observed in their healthy counterparts. Importantly, the serum concentration of Follistatin correlates significantly with tumor staging and microvessel density (MVD), reverting to physiologically balanced levels upon complete tumor resection (55). The anti-angiogenic multi-kinase inhibitor, sunitinib, has been substantiated for its efficacy in the treatment of refractory thymic carcinoma and thymoma as a first-line therapeutic option. Impressively, disease control, encompassing complete response, partial response, and stable disease, was achieved in 91% of thymic carcinoma cases and 86% of thymoma cases. Administration of sunitinib instigated an upregulation of CTLA-4 and PD-1, immune checkpoint molecules associated with improvements in overall survival (19). As a well-established anti-angiogenic agent, lenvatinib has demonstrated efficacy in various advanced cancers, including hepatocellular and thyroid carcinoma (56–58). The results from the phase 2 REMORA trial have provided the therapeutic effectiveness of lenvatinib in treating advanced thymic carcinoma patients. Among the 42 enrolled patients, 6 cases (38%) achieved a partial response, while 24 cases (57%) experienced stable disease. The median follow-up period was 15 months, resulting in an overall response rate of 38%. These findings underscore the potential of lenvatinib as a promising treatment option for advanced thymic carcinoma (59).

The proto-oncogene KIT encodes the type III receptor tyrosine kinase c-KIT, which plays a crucial role in several malignant neoplasms, including gastrointestinal stromal tumors (GISTs), chronic myeloid leukemia (CML), mastocytosis, melanoma, and germ cell tumors. KIT mutations and aberrant c-KIT expression are frequently observed in GISTs, with prevalent occurrences in exons 11, 9, 13, and 17. Noteworthy clinical observations indicate that patients harboring exon 11 mutations exhibit a heightened objective response rate to the tyrosine kinase inhibitor imatinib. Despite c-KIT expression being detected in a modest 0%-5% of thymomas and a substantial 50%-88% of thymic carcinomas, it does not consistently parallel the presence of KIT mutations. Remarkably, KIT mutations manifest in a mere 12.5% of cases and exclusively within those displaying positive c-KIT expression (60, 61). Thymic carcinomas harboring KIT mutations have been documented to display sensitivity to targeted therapeutics such as imatinib. Notably, mutations within exon 11, including V560del, V559G, Y553N, and L576P, exhibit discernible responsiveness to imatinib, while the H697Y mutation (in exon 14) demonstrates sensitivity to sunitinib. Likewise, the D820E mutation (in exon 17) and the K642E mutation (in exon 13) manifest sensitivity to sorafenib, whereas the 577-579del mutation (in exon 11) displays susceptibility to sorafenib treatment (62). Moreover, an intriguing observation emerges among approximately 70% of thymic squamous cell carcinomas, wherein all KIT-positive cases exhibit a distinctive tuft cell–like phenotype characterized by the robust expression of POU2F3, an event absent in thymomas. The remarkable correlation observed between POU2F3 and KIT expression implies the potential involvement of POU2F3 in the regulatory mechanisms governing KIT expression. The pronounced upregulation of POU2F3 represents a novel and enigmatic avenue within the carcinogenic repertoire (63).

CYLD, a ubiquitin-specific protease, serves as a key regulator involved in the intricate orchestration of the NF-ΚB signaling pathway. Notably, CYLD exerts its influence on cellular fate through modulating the ubiquitination state of receptor-interacting protein kinase 1 (RIPK1), thereby mediating programmed cell death. Pertinently, The loss of CYLD hinders the process of apoptosis by inducing the activation of NF-ΚB and facilitating the NF-ΚB-mediated transcriptional upregulation of prosurvival genes (64). Remarkably, CYLD assumes a pivotal role as a regulatory factor during T cell development, particularly during the transition from double-positive thymocytes to single-positive thymocytes. By eliciting proximal T cell receptor signaling, CYLD orchestrates the differentiation and maturation of thymic medullary epithelial cells, thus profoundly influencing the intricate process of negative selection within the thymus microenvironment (65). The prevalence of CYLD gene mutations in thymic carcinomas exceeds 10%. Investigations have unveiled that CYLD deficiency in TETs leads to an augmentation in programmed death-ligand 1 (PD-L1) expression, with a significant association between reduced CYLD expression and elevated PD-L1 levels. Notably, patients afflicted with advanced TETs exhibiting diminished CYLD expression fare worse in terms of prognosis compared to those with elevated expression. Considering the low tumor mutation burden characterizing TETs, heightened PD-L1 expression remains the most dependable biomarker for prognosticating the therapeutic efficacy of PD-1 antibodies in TETs. As such, PD-1/PD-L1 inhibitors hold potential as a therapeutic strategy for TETs patients displaying reduced CYLD expression (66).

The nucleocytoplasmic shuttling of proteins is a fundamental mechanism by which cells tightly regulate protein activity. Among the molecular players involved in this intricate process, exportin 1 (XPO1) serves as a pivotal mediator, facilitating the transport of numerous tumor suppressor proteins and oncoproteins from the nucleus to the cytoplasm. Aberrant expression of XPO1 disrupts the function of critical tumor suppressors, including p53, IΚB, p27, and FOXO3A. Notably, dysregulation of XPO1 has been reported in leukemia and various solid tumors. Insightfully, heightened XPO1 expression has been observed in aggressive and advanced-stage TETs, correlating with an unfavorable prognosis (67). Selinexor, a potent and selective inhibitor of XPO1, has been revealed to instigate its proteasomal degradation. This biochemical event facilitates the nuclear retention and subsequent activation of key tumor suppressor proteins, curtails the translation of oncoprotein mRNA, and promotes cell cycle arrest and apoptosis in a broad spectrum of hematological malignancies and solid tumors (68, 69). Specifically, within the context of TET cells, Selinexor governs the nuclear accumulation of the tumor suppressor proteins FOXO3a, p53, and p27. Furthermore, Selinexor mediates cell cycle arrest through the modulation of a multitude of proteins that orchestrate cell cycle progression and apoptosis by inducing the expression of pro-apoptotic proteins such as BIM and BAX. It is noteworthy that XPO1 also targets GTF2I. In a Phase I clinical trial aiming to assess the safety and efficacy of Selinexor, a cohort of 189 patients with advanced solid tumors was evaluated. Critically, within this cohort, we included four patients diagnosed with TETs. Outcomes included one patient achieving a partial response and three patients exhibiting stable disease. These data collectively implicate Selinexor as a promising candidate for the therapeutic armamentarium against TETs in the future (67, 70).

## Genetic rearrangements

Metaplastic thymoma, a distinctive subtype of TETs, manifests unique molecular characteristics that set it apart from other types of thymomas. Notably, the recurrent YAP1-MAML2 fusion has emerged as a defining molecular alteration in metaplastic thymoma, playing a pivotal role in its pathogenesis. In cellular models encompassing ovarian cancer and glioblastoma, the YAP1-MAML2 fusion generates a chimeric protein that activates YAP1-related transcriptional programs through a TEAD1-dependent mechanism, thereby promoting tumor cell proliferation (71, 72). Additionally, KMT2A-MAML2 translocation has been observed in 6% of clinically invasive type B2 and B3 thymomas, as well as in one case of combined thymic carcinoma (type B3 thymoma with small thymic carcinoma component). Noteworthy for its potent oncogenic activity upon fusion with partner genes in sarcomas and leukemias, the KMT2A gene exhibits conspicuous carcinogenic potential in such rearrangements (73, 74). Furthermore, the CRTC1-MAML2 fusion has been identified in 56% of thymic mucoepidermoid carcinomas, exhibiting an association with lower clinical stages and improved overall survival rates (75). The detection of these diverse gene fusions underscores the potential utility of MAML2 gene rearrangements as prospective biomarkers for the morphological classification of thymomas. While the precise functional implications of these fusions in metaplastic thymoma remain elusive, they are postulated to function as pivotal oncogenic drivers, warranting further investigation.

## Epigenetic modifications

**Figure 2:**
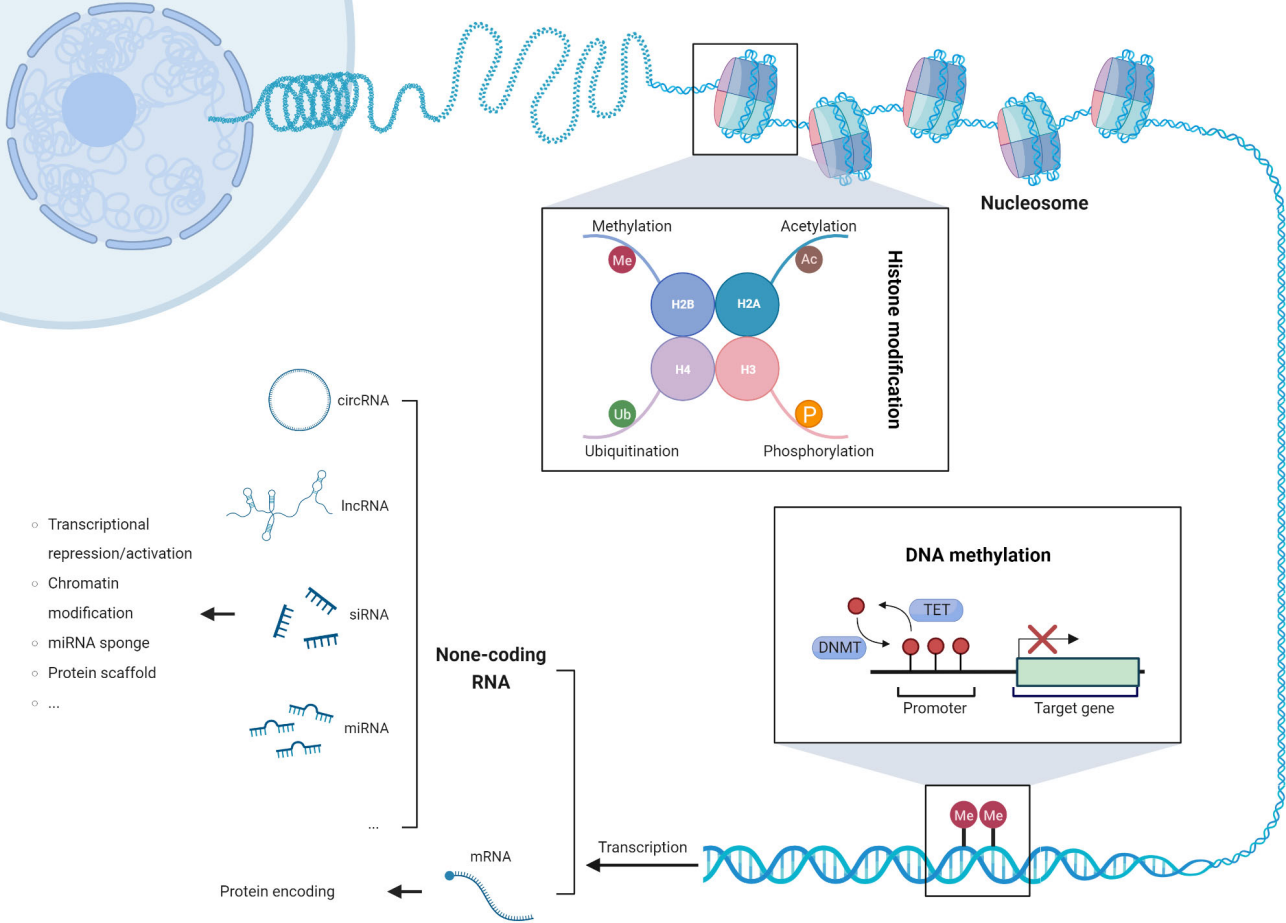
The three mechanisms of epigenetic modifications: DNA methylation, post-translational modification of histone tails, and dysregulated expression of non-coding RNAs.

Epigenetic modifications, such as DNA methylation changes, aberrant expression of non-coding RNAs, and post-translational modifications of histone tails, have emerged as pivotal drivers of genomic instability and chromosomal aberrations in cancer cells (**Figure 2**). These modifications intricately modulate the gene expression landscape, activating transposable elements, upregulating oncogenes, and silencing tumor suppressor genes, thereby fostering the onset and progression of tumorigenesis (76). Remarkably, a subset of epigenetic genes implicated in chromatin remodeling (e.g., SMARCA4), histone modifications (e.g., BAP1, SETD2, ASXL1), and DNA methylation (e.g., TET2, DNMT3A, WT1) exhibits recurrent somatic mutations. Notably, these mutations occur at varying frequencies between thymic carcinoma and thymoma subtypes. In thymic carcinoma, the prevalence of these mutations reaches 38%, surpassing the observed frequency of 10% in thymoma (36). This distinct pattern highlights the potential association between specific epigenetic alterations and the aggressive behavior of thymic carcinoma. Understanding the role of epigenetic modifications and their impact on tumor biology is crucial for elucidating the underlying mechanisms of thymic tumorigenesis. Further investigations are warranted to decipher the functional consequences of these somatic mutations in chromatin remodeling, histone modifications, and DNA methylation. Additionally, exploring the clinical implications of these epigenetic alterations may pave the way for the development of targeted therapeutic approaches and personalized treatment strategies in thymic tumors.

DNA methylation is involved in a series of cellular and biological processes, including cell differentiation, aging, tissue-specific gene expression, genomic stability, and genomic imprinting. Apart from its significance in normal developmental processes, DNA methylation also plays a role in pathology, such as carcinogenesis (77). The TCGA dataset analysis uncovered a comprehensive landscape of CpG site tgfmethylation alterations in TETs. Among the identified sites, 5155 exhibited heightened methylation while 6967 displayed diminished methylation. Notably, approximately 3600 of these sites were situated within gene promoters, indicating their regulatory significance. Hypermethylation within these pivotal regions resulted in the transcriptional repression of 134 genes, while 174 genes exhibited an increase in mRNA expression. Furthermore, the Cox regression analysis revealed a significant correlation between the methylation levels of 187 loci in TET patients and overall survival. Remarkably, specific independent prognostic factors, including cg05784862 (KSR1), cg07154254 (ELF3), cg02543462 (ILRN), and cg06288355 (RAG1), were identified (78). In addition, DNA hypermethylation-mediated gene silencing affected a diverse array of target genes, encompassing CDH1, CDKN2A, FHIT, MGMT, and MLH1 (79). Notably, the methylation pattern of the tumor suppressor gene MGMT exhibited a higher frequency in thymic carcinoma compared to thymoma, correlating with advanced disease stages and increased sensitivity to alkylating agents (80). Furthermore, the overexpression of METTL3 was observed in tumor tissue compared to its normal counterpart. Silencing METTL3 expression in thymic carcinoma cells led to decreased cell proliferation and reduced overall translation efficiency. Notably, METTL3 played a pivotal role in driving c-MYC expression in TET cells. Specifically, the upregulation of c-MYC protein was facilitated by the methylation-dependent delocalization of the long non-coding RNA MALAT1, a process orchestrated by METTL3 (81). These findings shed light on the complex interplay between DNA methylation, gene regulation, and tumorigenesis in thymic tumors, underscoring the potential therapeutic implications of targeting METTL3 in this context. In the context of Thymoma-associated myasthenia gravis (TAMG), an intriguing observation emerges regarding the methylation patterns of the MTHFR and DNMT3A promoters in tumor tissues. Notably, these genomic regions display significantly elevated levels of methylation within the tumor samples compared to blood samples. Furthermore, the methylation status of MTHFR within tumor tissues exhibits a remarkable increase when compared to healthy neighboring thymic epithelial cells (82). Moreover, a noteworthy study conducted by Yan et al. reveals an intriguing inverse correlation between MTHFR methylation and its corresponding expression levels in TETs. The authors speculate that the diminished activity of the MTHFR enzyme, influenced by the MTHFR C667T gene polymorphism, may contribute to significant DNA hypomethylation in TETs, thereby promoting the activation of crucial oncogenes (83).

Non-coding RNA alterations have emerged as pivotal determinants in the intricate landscape of TETs, playing a multifaceted role in their development and progression. MicroRNAs (miRNAs) and long non-coding RNAs (lncRNAs) stand at the forefront, orchestrating a complex interplay of molecular mechanisms as both oncogenes and tumor suppressors. Through amplification, deletion, aberrant epigenetic modifications, and transcriptional regulation, these non-coding RNA species wield their influence with precision. Notably, discernible disparities in miRNA expression profiles exist not only between TET and normal tissues but also across distinct histological subtypes of TET. A comprehensive understanding of these non-coding RNA alterations holds the promise of unraveling the underlying pathogenesis of TET, paving the way for targeted therapeutic interventions in the future.

In TETs, there is a notable upregulation of miRNA-21-5p and a downregulation of miRNA-145-5p. Of particular significance, miRNA-145-5p is recognized as a prominent tumor suppressor and exhibits a reciprocal relationship with the expression of EGFR. The expression of miRNA-145-5p is tightly controlled by epigenetic modifications. Intriguingly, investigations have demonstrated that the administration of epigenetic modulators can induce the expression of miRNA-145-5p in TET cells, resulting in the downregulation of its target genes and enhancing its anti-tumor properties (84). The dysregulation of specific miRNAs, such as miRNA-142-5p, miRNA-363-3p, and miRNA-16-2-3p, has emerged as a noteworthy phenomenon in thymic carcinoma, exerting potential regulatory roles in pivotal molecular pathways encompassing BIRC3, SCYA20, and MYC-associated pathways (85). Remarkably, a miRNA cluster located on chromosome 19q13.42 exhibits prominent overexpression in type A and AB thymomas, leading to the activation of the PI3K/AKT/mTOR signaling cascade. In a fascinating contrast, this miRNA cluster experiences transcriptional repression attributed to promoter methylation in thymic carcinoma (30). Conversely, a notable reduction in the expression of the miRNA cluster on chromosome 14q32 has been observed in thymic carcinoma compared to type A thymomas (33). Moreover, an extensive analysis has uncovered intriguing associations between specific miRNAs and overall survival in thymomas. Notably, miRNA-140, miRNA-450b, miRNA-542, miRNA-639, miRNA-3613, and miRNA-3913-1 exhibit a positive correlation with overall survival, suggesting their potential as prognostic indicators in thymomas. Conversely, miRNA-1976 displays a negative correlation with overall survival, underscoring its potential utility as a prognostic biomarker in this context (86).

The dysregulation of lncRNAs has emerged as a crucial factor in tumor progression. In the context of TETs, notable associations have been uncovered between specific lncRNAs and key molecular players. For instance, an upregulation of LOXL1-AS1 has been observed, which correlates positively with HSPA9 expression, while concomitantly exhibiting a concordant downregulation of miR-525-5p. Remarkably, miR-525-5p acts as a tumor suppressor by restraining cell growth and invasion through the targeted inhibition of HSPA9, consequently inducing apoptosis. Acting as a molecular sponge for miR-525-5p, LOXL1-AS1 amplifies HSPA9 expression, thereby facilitating the progression of thymic carcinoma (87). Similarly, LINC00174 serves as a molecular sponge for miR-145-5p, thereby promoting the expression of SYBU, FEM1B, and SCD5 genes. Among these genes, SCD5, one of the target genes of LINC00174, plays a role in lipid metabolism control and enhances the migration of thymic carcinoma cells (88). The differential expression of lncRNAs exerts regulatory control over diverse biological processes and molecular pathways, thereby positioning them as potential prognostic factors and therapeutic targets for patients (89). Moreover, in the context of TAMG, lncRNAs exhibit specific expression patterns. Notably, XLOC_003810 promotes the activation of CD4+ T cells and inflammatory factors, including IFN-γ, thereby regulating the delicate balance between Th17 and Treg cells (90, 91). Furthermore, immune-related lncRNAs, such as AC004943.1, FOXG1-AS1, and WT1-AS, display elevated expression levels in TAMG patients due to their hypomethylation status, implicating their involvement in the mechanisms underlying TAMG pathogenesis (92).

Post-translational modifications of histones play a pivotal role in the dynamic regulation of gene expression, exerting a profound influence on the interplay between DNA and histone proteins. These modifications, including acetylation, methylation, and ubiquitination, modulate chromatin structure and function, ultimately impacting transcriptional processes. Among these modifications, acetylation has been extensively studied for its role in enhancing gene expression by neutralizing the positive charge of histones and promoting a more relaxed chromatin state. On the other hand, methylation of histones can either activate or repress gene expression depending on the specific residues and context involved. Furthermore, ubiquitination serves as a versatile modification that regulates various cellular processes, including DNA repair, transcriptional activation, and protein degradation (93). One intriguing area of research in the field of histone modifications is the development of therapeutic strategies targeting these epigenetic marks. Notably, the histone deacetylase inhibitor belinostat has emerged as a promising candidate for cancer treatment. Belinostat has demonstrated remarkable efficacy, particularly when combined with the PAC chemotherapy regimen, in patients with thymoma and thymic carcinoma. The objective response rates observed in these cohorts highlight the potential of targeting histone deacetylases as a viable therapeutic approach for these malignancies. In addition to its direct impact on chromatin remodeling and gene expression, belinostat has also been found to possess immunomodulatory properties. Studies have revealed that belinostat treatment leads to a reduction in regulatory T cells and exhaustion of CD8(+) T cells in the peripheral blood of patients. This observation suggests a potential link between epigenetic modifications and the immune microenvironment in thymic epithelial tumors (94).

Epigenetic modifications play a crucial role in the development and progression of thymic tumors. Dysregulation of DNA methylation, non-coding RNAs, and histone modifications leads to aberrant gene expression patterns, contributing to genomic instability, tumor suppressor gene silencing, and oncogene activation. Understanding the functional consequences of these epigenetic alterations is essential for elucidating the underlying mechanisms of thymic tumorigenesis and identifying potential therapeutic targets. Furthermore, integrating epigenetic biomarkers into clinical practice may facilitate personalized treatment approaches for patients with thymic tumors.

## The tumor microenvironment

In the field of oncology, there has been a growing interest in investigating the tumor microenvironment (TME) and its potential response to ICIs targeting the PD1/PD-L1 interaction. Similar to highly heterogeneous tumors such as hepatocellular carcinoma and melanoma, delving into the complexities of the TME plays a pivotal role in assessing prognostic outcomes and the efficacy of immunotherapeutic interventions (95, 96). The components of the TME exert diverse influences on tumor progression and anti-tumor immune responses. Within the TME, dominant cellular subpopulations encompass diverse leukocyte lineages, including myeloid and lymphoid origins. Certain immune cells exert tumor-suppressive functions, while others act in a permissive manner, thus facilitating the coexistence of pro-tumoral inflammation and anti-tumor immune responses, establishing a dynamic equilibrium within the TME. The abundance and activation status of different cell populations determine the direction of this delicate balance. Firstly, T lymphocytes exhibit distinct levels of maturity and functional states across various histological subtypes, establishing themselves as the most abundant cellular subset in the TET tumor microenvironment. Notably, in type A, AB, B1, and B2 thymomas, a higher prevalence of immature T lymphocytes displaying dual positivity for CD4 and CD8 immune markers is observed. Conversely, type B3 thymomas and thymic carcinomas showcase a substantial infiltration of late-stage differentiated T cells, predominantly featuring either CD4 or CD8 single positivity, with a notable polarization towards a CD8 cytotoxic phenotype (97). Notably, research has revealed a compelling finding in type B3 thymomas and thymic carcinomas with CD4 and CD8 single-positive T cell characteristics. The administration of anti-PD-1 antibodies has been shown to enhance T cell cytokine production and cytotoxicity in these tumors, suggesting that type B3 thymomas and thymic carcinomas represent promising targets for anti-tumor immune therapy (98). Additionally, B cells demonstrate a subtype-specific distribution pattern, exhibiting a prominent enrichment in micronodular thymic neoplasms with follicular lymphoid hyperplasia. Within this subset, B cell presence is observed in micronodular thymoma with lymphoid B cell hyperplasia, as well as in micronodular carcinoma with lymphoid hyperplasia where they assemble into lymphoid aggregates with germinal centers (97). Dendritic cells, as specialized antigen-presenting cells in the immune system, play a crucial role in orchestrating the effective priming of CD8 T cells and generating soluble paracrine factors to recruit T cells into TME, thereby enhancing local cytotoxicity (99). Tumor-associated macrophages (TAMs) assume a crucial role within the tumor microenvironment, exerting dual influence on immunosuppression and tumor progression. These macrophages release a diverse repertoire of factors, including epidermal growth factor (EGF) and TGF-Β1, orchestrating cellular proliferation, survival, and extracellular matrix degradation, thereby facilitating invasive potential (100).

Moreover, TGF-Β acts as a pivotal player in the intricate immunoregulatory network. Its overexpression in advanced thymic carcinoma patients indicates its potential role in the pathogenesis of this malignancy, exerting negative modulation on cytotoxic CD8+ cells and promoting the activation of CD4+ regulatory T cells, thereby fostering self-tolerance and facilitating tumorigenesis (101). Previous research has identified that TGF-Β released from platelets plays a role in inhibiting NK cell activity upon intravital injection, contributing to distant metastasis (102). Furthermore, TETs exhibit distinct expression profiles of heat shock proteins, such as HSP27 and HSP70, which play a pivotal role in driving tumor progression through their pro-inflammatory and anti-apoptotic functions. Notably, a progressive reduction in the immunohistochemical expression of these heat shock proteins is observed across the spectrum from type A thymomas to thymic carcinomas, diverging from the trends observed in other malignancies. Conversely, an inverse correlation is observed in the serum concentration levels of these proteins. These intriguing findings position heat shock proteins as potential serum biomarkers for disease monitoring and promising targets for future immunotherapeutic approaches, particularly in conjunction with other therapeutic regimens (103). In addition, immunohistochemical analysis has confirmed the elevated expression of fibronectin B-domain in the stromal cells of TME, with particularly abundant expression observed in type B3 thymomas. The induction of fibronectin by thymoma-associated stromal cells leads to its transformation into the ED-B isoform, which represents a crucial step in tumor progression and metastasis (104).

In the field of oncology, the TET microenvironment and its potential responses to ICIs targeting PD1/PD-L1 interactions have recently garnered widespread attention. Immunohistochemical investigations have revealed the widespread presence of PD-L1 in the majority of TETs. Padda et al. reported PD-L1 overexpression in 68% of 69 TET patients (105). Similarly, Katsuya et al. demonstrated high PD-L1 expression in 70% of thymic carcinomas and 23% of thymomas within their cohort of 141 TET specimens (106). Consistently, Yokoyama et al. observed an elevation in PD-L1 expression in 80% of thymic carcinoma samples (107). However, the prognostic implications of PD-L1 expression remain contentious (105–107). Notably, PD-L1 and TMB have emerged as pivotal predictive factors for the response to ICIs (15). Giaccone et al. reported a modest response rate of 22.5% for the ICI pembrolizumab in thymic carcinoma, albeit accompanied by significant autoimmune toxicity affecting 15% of patients (108). Similarly, Cho et al. investigated the therapeutic efficacy of pembrolizumab in a cohort of 33 TET patients (comprising 7 thymomas and 26 thymic carcinomas), with an overall response rate of 21.2% (109).

Nevertheless, the use of ICI therapy in TETs has been associated with the emergence of severe toxic reactions, notably myocarditis, myositis/myalgia, transaminitis, and myasthenia gravis. In the context of thymic carcinoma, the administration of ICIs as monotherapy has shown a substantial occurrence rate of severe immune-related adverse events (IRAEs), ranging from 15% to 20% (108, 109). However, when avelumab and axitinib are combined, a relatively more tolerable profile of AEs is observed, with 12% of patients experiencing severe IRAEs, including instances of pneumonia and polymyositis (110). For patients with thymoma, the utilization of ICIs has been linked to a higher incidence (ranging from 38% to 71.4%) of severe and occasionally fatal immune-mediated toxic reactions (109, 111). Importantly, compared to other solid tumors treated with ICIs, TETs exhibit a distinct propensity for multi-organ involvement and treatment-resistant IRAEs. Consequently, a judicious approach to immune-based therapies is warranted in this patient cohort, even in cases of thymic carcinoma.

Studies investigating the potential of ICIs in TETs have examined PD‐L1 expression and genomic alterations as putative biomarkers for treatment response. Elevated PD-L1 expression has shown a positive correlation with a more favorable response to pembrolizumab (108, 109). Conversely, in patients with B3 thymoma and thymic carcinoma, although PD-L1 expression correlates positively with higher tumor TMB, it does not significantly impact the response rate to avelumab and axitinib (110). Noteworthy differences have been observed between patients with high PD‐L1 expression and those with low or no PD-L1 expression, where the latter group is more likely to harbor TP53 mutations, while patients with CYLD mutations exhibit elevated PD-L1 expression (108). Peripheral blood mononuclear cell analysis revealed that patients responding to avelumab have higher absolute lymphocyte counts and lower frequencies of B cells, regulatory T cells, conventional dendritic cells, and natural killer cells (111). These findings highlight the significance of immune cell characteristics both in the tumor microenvironment and peripheral blood. It is imperative to develop biomarkers for predicting the risk of IRAEs to ensure the judicious use of ICIs in this high-risk patient population.

In addition to PD-L1, Arbour et al. identified other immune co-stimulatory and co-inhibitory markers, such as TIM-3, CTLA-4, GITR, ICOS, and CD137, exhibiting high expression within the TET tumor microenvironment. These findings provide potential avenues for targeted therapeutic interventions (112).

Myasthenia gravis (MG) is an autoimmune disorder characterized by the production of self-directed antibodies against diverse targets at the neuromuscular junction. Approximately 30-40% of thymomas occur in patients with a specific type of MG known as thymoma-associated MG (TAMG) (113). The pathogenesis of TAMG hinges crucially on alterations in the tumor immune microenvironment, which disrupt central tolerance mechanisms and impede immune regulation. In particular, defective expression of the autoimmune regulator (AIRE) and the forebrain-expressed zinc finger 2 (Fezf2) has been observed in the majority of thymomas, potentially contributing to compromised negative selection of self-reactive T cells, as well as defective positive selection of immunosuppressive central regulatory T cells, thereby offering a partial explanation for the link between autoimmune diseases and thymomas (114). Apart from the downregulation of AIRE and Fezf2, other pivotal mechanisms in central immune tolerance disruption and predisposition to autoimmunity include perturbation of the normal thymic architecture and loss of expression of major histocompatibility complex (MHC) class II molecules in thymoma cells. Immune dysregulation fosters an abundance of lymphocytes in an immature state, which may serve as a reservoir for self-reactive cells upon entry into the bloodstream (115). TAMG is characterized by a microenvironment that facilitates the generation of autoantibodies, encompassing the formation of ectopic germinal centers, the accumulation of T follicular helper cells, and the migration of type 2 conventional dendritic cells (116). Transcriptomic analysis within the tumor revealed the upregulation of genes encoding medium-sized neurofilament (NEFM) and ryanodine receptor type 3 (RYR3), which share sequence similarities with major antigenic targets implicated in myasthenia gravis, such as acetylcholine receptor (AChR), titin, and ryanodine receptor type 1 and 2 (RYR1, RYR2). These findings suggest a potential role of a “molecular mimicry” mechanism in the development of myasthenia gravis in TAMG. Furthermore, autoimmune syndromes in TAMG patients may be linked to specific genomic alterations, as evidenced by a higher rate of aneuploidy (15).

Gaining a comprehensive understanding of the composition and dynamics of the TME not only sheds light on the mechanisms driving tumor progression but also reveals potential targets for therapeutic interventions. Moreover, this knowledge paves the way for the development of personalized treatment strategies tailored to the unique characteristics of individual TET patients. Thus, investigating the TME in TETs represents a crucial avenue for advancing our comprehension of these tumors and ultimately improving patient outcomes.

## Conclusion

In this comprehensive review, we delve into the intricate landscape of TETs, covering chromosome abnormalities, molecular subtypes, gene mutation abnormalities, gene expression dysregulation, genetic rearrangements, epigenetic abnormalities, and the tumor microenvironment. Our synthesis of current knowledge and research findings significantly contributes to the understanding of TETs and underscores their clinical significance. The study of TETs holds paramount importance due to their rarity and heterogeneous clinical behavior. By unraveling the underlying mechanisms and molecular characteristics, we can enhance diagnostic precision, prognostic stratification, and personalized treatment modalities for TET patients. This review serves as a pivotal resource, consolidating existing evidence and catering to the needs of researchers, clinicians, and oncologists immersed in this field.

While this review presents a comprehensive synthesis of the molecular characteristics of TETs, it is essential to acknowledge the limitations inherent in the field and this review. The rarity of TETs inherently restricts the sample sizes of the discussed studies, potentially impacting the generalizability of certain findings. Additionally, we have not delved into potential discrepancies and conflicting results present in the literature, which may stem from differences in study design, methodologies utilized, or the inherent heterogeneity of TETs themselves. It is pertinent to note that, while encompassing a broad spectrum of molecular features, from chromosomal abnormalities to epigenetic changes, further exploration is needed to understand the relative importance and interplay of these features. Moreover, the strength of evidence varies across topics, with certain areas, such as the role of non-coding RNAs, necessitating additional studies for confirmation. Lastly, while touching upon potential therapeutic implications, the translation of these molecular insights into clinical practice is a complex process. Identifying robust therapeutic targets and devising effective treatment strategies remain daunting tasks. Integrating knowledge of genetic abnormalities, gene expression dysregulation, and the intricate interplay with the tumor microenvironment will be pivotal for designing novel therapeutic approaches. Multidisciplinary collaborations, encompassing oncology, pathology, genomics, immunology, and bioinformatics, will be instrumental in surmounting these challenges. Looking ahead, future TET research should prioritize several key areas. Large-scale collaborative endeavors are imperative to establish comprehensive datasets and bolster the statistical power of studies. Additionally, integrating multi-omics approaches, including genomics, transcriptomics, and epigenomics, can provide a holistic comprehension of TET biology, facilitating the identification of potential therapeutic targets. Furthermore, developing preclinical models that faithfully recapitulate TET characteristics will expedite the evaluation of novel treatment strategies and bridge the translational gap. For example, nomograms play a crucial role in predicting clinical outcomes in cancer, aiding in the enhancement of survival prognosis, and subsequently generating rational treatment strategies and recommendations (117–119).

In summary, this review sheds light on the unique molecular landscape of TETs, emphasizing the significance of unraveling TETs for refined diagnostics, prognostication, and therapeutic interventions. By addressing current challenges and embracing future research directions, significant advancements can be achieved in the field of TETs, ultimately enhancing patient outcomes and quality of life.

## Author contributions

XZ: Conceptualization, Formal Analysis, Investigation, Project administration, Writing – original draft. PZ: Conceptualization, Formal Analysis, Investigation, Project administration, Writing – original draft. AC: Data curation, Resources, Writing – original draft. YF: Conceptualization, Validation, Writing – original draft. HC: Writing – review & editing. ZX: Writing – review & editing. HT: Investigation, Writing – review & editing.
